# A Flexible 12-Lead/Holter Device with Compression Capabilities for Low-Bandwidth Mobile-ECG Telemedicine Applications

**DOI:** 10.3390/s18113773

**Published:** 2018-11-05

**Authors:** Flavio Pineda-López, Andrés Martínez-Fernández, José Luis Rojo-Álvarez, Arcadi García-Alberola, Manuel Blanco-Velasco

**Affiliations:** 1 Department of Signal Theory and Communications, Telematics and Computing Systems, Rey Juan Carlos University, 28943 Madrid, Spain; andres.martinez@urjc.es (A.M.-F.); joseluis.rojo@urjc.es (J.L.R.-Á.); 2 Departamento de Eléctrica y Electrónica, Universidad de las Fuerzas Armadas ESPE, Av. General Rumiñahui s/n, Sangolquí 171-5-231B, Ecuador; 3 Center for Computational Simulation, Universidad Politécnica de Madrid, Boadilla, 28223 Madrid, Spain; 4 Cardiology Service, Arrhythmia Unit, Hospital General Universitario Virgen de la Arrixaca, El Palmar, 30120 Murcia, Spain; arcadi@secardiologia.es; 5 Signal Theory and Communications Department, Alcalá University, 33600 Madrid, Spain; manuel.blanco@uah.es

**Keywords:** ECG, Holter, Android™, telemedicine, signal compression low-bandwidth, rural areas, STM32F microcontroller

## Abstract

In recent years, a number of proposals for electrocardiogram (ECG) monitoring based on mobile systems have been delivered. We propose here an STM32F-microcontroller-based ECG mobile system providing both long-term (several weeks) Holter monitoring and 12-lead ECG recording, according to the clinical standard requirements for these kinds of recordings, which in addition can yield further digital compression at stages close to the acquisition. The system can be especially useful in rural areas of developing countries, where the lack of specialized medical personnel justifies the introduction of telecardiology services, and the limitations of coverage and bandwidth of cellular networks require the use of efficient signal compression systems. The prototype was implemented using a small architecture, with a 16-bits-per-sample resolution. We also used a low-noise instrumentation amplifier TI ADS1198, which has a multiplexer and an analog-to-digital converter (16 bits and 8 channels) connected to the STM32F processor, the architecture of which incorporates a digital signal processing unit and a floating-point unit. On the one hand, the system portability allows the user to take the prototype in her/his pocket and to perform an ECG examination, either in 12-lead controlled conditions or in Holter monitoring, according to the required clinical scenario. An app in the smartphone is responsible for giving the users a friendly interface to set up the system. On the other hand, electronic health recording of the patients are registered in a web application, which in turn allows them to connect to the Internet from their cellphones, and the ECG signals are then sent though a web server for subsequent and ubiquitous analysis by doctors at any convenient terminal device. In order to determine the quality of the received signals, system testing was performed in the three following scenarios: (1) The prototype was connected to the patient and the signals were subsequently stored; (2) the prototype was connected to the patient and the data were subsequently transferred to the cellphone; (3) the prototype was connected to the patient, and the data were transferred to the cellphone and to the web via the Internet. An additional benchmarking test with expert clinicians showed the clinical quality provided by the system. The proposed ECG system is the first step and paves the way toward mobile cardiac monitors in terms of compatibility with the electrocardiographic practice, including the long-term monitoring, the usability with 12 leads, and the possibility of incorporating signal compression at the early stages of the ECG acquisition.

## 1. Introduction

A number of physiological systems in the human body generate a wide variety of signals with different natures, the monitoring of which can provide us with highly useful information about their usual functioning and changes in health state. Among the most widely known of these signals are the bioelectric potentials associated with muscle activity and the nerve conduction potentials [1]. In order to monitor the signals generated by the cardiac muscles, many systems have been developed to support the diagnosis and treatment of patients with heart diseases, which are intended either for medical practice or for disease research. Several techniques have been recently developed to improve the acquisition and to display the electrocardiogram (ECG) signals, and are increasingly focused on use on cellphones, and they are called here mobile ECG systems. However, these proposals sometimes correspond to partial solutions in terms of the available mobile technology at that moment. Current electrocardiography often works in two ways, namely, with the recording of a 12-lead ECG during 10–20 s and with the recording of 2–3 leads during 24 h in the so-called Holter monitoring, and this last one is currently being extended to 7-day and longer Holter monitoring. Mobile ECG systems so far only partially achieves some of these applications. In addition, it is usual that signal processing, both in conventional and in mobile ECG systems, is made in stages close to the application stage, and not close to the recording and pre-sending stage. However, compression processing in the later ones would be an interesting option in some cases. And finally, long-term monitoring in current ECG mobile systems is often constrained by memory issues, by transmission issues, or by both.

A scenario that requires specific solutions is that of health care facilities in rural areas of developing countries. The lack of medical specialists and the limited bandwidth offered by cellular networks in these areas make it necessary to design telemedicine systems specially adapted to this reality [2]. Although the number and configuration of leads or the recording duration can be improved in existing systems, both characteristics may not be jointly necessary, as a 12-lead ECG is usually taken during some few seconds in controlled conditions, whereas a Holter requires 2–3 leads for some hours or days in ambulatory conditions. Nevertheless, a mobile system providing both of them can have advantages in some aspects such as simplicity or versatility. According to the previous considerations, we propose here a prototype of an ECG mobile system, based on the STM32F microcontroller, which provides us with long-term Holter monitoring and high-resolution 12-lead ECG recording, and this same architecture also enables us to make efficient compression processing close to the acquisition stages. In our implementation, the prototype measures the ECG signals from 10 electrodes on the patient body and generates the 12 ECG leads with simple mathematical processing. Alternatively, it can measure 2 or 3 leads with a different set of cables in a patient during several days. This data is sent via Bluetooth to the cellphone to be displayed and then to a web server for remote monitoring. The system allows the real-time execution of compression algorithms using filter banks as an implementation example, and it also allows the system to send the ECG information through narrow communication channels. The use of this lossy compression algorithm has shown the ability to considerably reduce the required bandwidth (especially useful in rural areas of developing countries), and the quality of the ECG signals subject to compression has been checked in the present work to be clinically valid. On the other hand, Holter devices usually work with two or three leads, but it could still be advantageous to use a flexible single system capable of doing both a 12-lead ECG and a 2- or 3-lead Holter by simply changing the the set of cables, rather than acquiring two separate devices, as long as such a design does not degrade the system characteristics, such as weight, size, price, or battery consumption.

This paper is organized as follows. Section 2 summarizes a compendium of related works in the field of mobile systems previously proposed for ECG monitoring, which can be seen as related precedents of the current proposal. Section 3 provides the description of the general architecture of the proposed and developed prototype, which includes the acquisition and storage modules, the cellphone, and the web server. Section 4 presents the general software architecture, emphasizing the acquisition software, the cellphone software, and the web server software. Section 5 shows several performance tests (storage, power consumption, data quality, compression rate, and calibration with respect to commercial electrocardiographs). Section 6 gives the results with recordings from simulators and patients when evaluated by the clinical staff. Finally, Section 7 summarizes the conclusions.

## 2. Previous Related Works

An increasing number of ECG-recording mobile systems is emerging every year. Several of these proposed systems in the literature used 3 leads taken from the patient to generate the 12 leads by subsequently using processing techniques in the cellphone, but the obtained ECG sometimes exhibited waveform distortion and time cuts on the rebuilt signals [3]. Many projects have used Arduino™ modules for signal processing, and the data were then sent to the cellphone, so that the patients could observe their ECG signal in real time at any place [4], though often limited to one single lead. Applications for Android™-based mobile devices have been developed for real-time ECG monitoring and automated arrhythmia detection by analyzing the ECG parameters from a single lead [5]. The Advanced RISC Machine (ARM) microprocessor STM32A was used to implement a telecardiology system using a mobile 7-lead ECG device, and to provide a 24 h health monitoring service. The use of 7-lead wires has been proposed to help collect enough ECG data to guarantee the detection accuracy without impairing the system mobility [6]. This same idea was extended [7] to incorporate a system-on-chip in the prototype, the so-called CardioChip, for the acquisition of ECG signals, which was a single-channel, low-power, small-size ECG application-specific integrated circuit, designed for personal mobile applications. In addition, the algorithms developed in a personal computer (PC) for R-peak detection and respiratory rate analysis provided with satisfactory sensitivity and precision, according to the authors.

Current mobile technology is at an increasingly mature stage, hence it offers new possibilities. Commercial discrete components, such as on-chip operation amplifier MSP430FG439 and the Bluetooth system-on-chip CC2540, were used in the portable ECG prototype in [8], where a cellphone based on an Android™ system provided a communication gateway. Therefore, the ECG data could be easily transferred to the remote doctors via 3G-mobile communication networks with 1 KB/s bandwidth. Other prototypes have used a Peripheral Interface Controller (PIC) for ECG monitoring using an Android™ mobile phone and Bluetooth. In [9], one single lead was digitized with a 10-bit Analog-to-Digital Converter (ADC), processed in the PIC, and sent to the cellphone for viewing. Another basic ECG mobile system was recently implemented with the filtering and conditioning modules for a single lead with instrumentation and operational amplifiers [10]. The ECG signal digitization was made with an Arduino™ board, and this signal was sent to the cellphone via Bluetooth. Multiple-purpose ECG acquisition systems have also been designed to analyze the stress, often considering a single lead and processing the signals in the cellphone [11]. The use of basic microcontrollers of the STMicroelectronics™ family in ECG prototypes is common nowadays [12]. In [13], a single-lead portable ECG prototype was implemented by using the STM32L476xx microcontroller, which was used for control purposes and to send the ECG data to the user. The NXPLPC1768 microcontrollers of the ARM series were used to design a 12-lead wireless ECG device using the TI ADS 1198 front-end amplifier [14] and to visualize the data in the PC without using a cellphone [15].

Many innovative projects applied to remote sites face difficulties that are due to the communication availability at the rural areas is not constant [16]. In rural areas, the coverage of cellular networks is very poor and many problems arise there for allowing robust and secure communications. It has been estimated that 89% of the world’s urban population will have third generation (3G) or Long Term Evolution (LTE) coverage by the end of 2019, whereas the situation is different for the rural population, as only 29% are expected to have 3G coverage by that date [17].

Table 1 shows a detailed performance evaluation in terms of the number of leads, the use of a cellphone, the implementation of parameter detection subsystem, the use of Internet cloud, compression capacity, noise, input range, power consumption, CMRR, noise, input impedance, and sampling rate. These parameters allow a direct comparison with state-of-the-art prototypes and systems recently proposed in the literature, as well as a justification for some of the features addressed by our system.

## 3. Hardware Architecture of the Flexible 12-Lead/Holter Prototype

**Figure 1:**
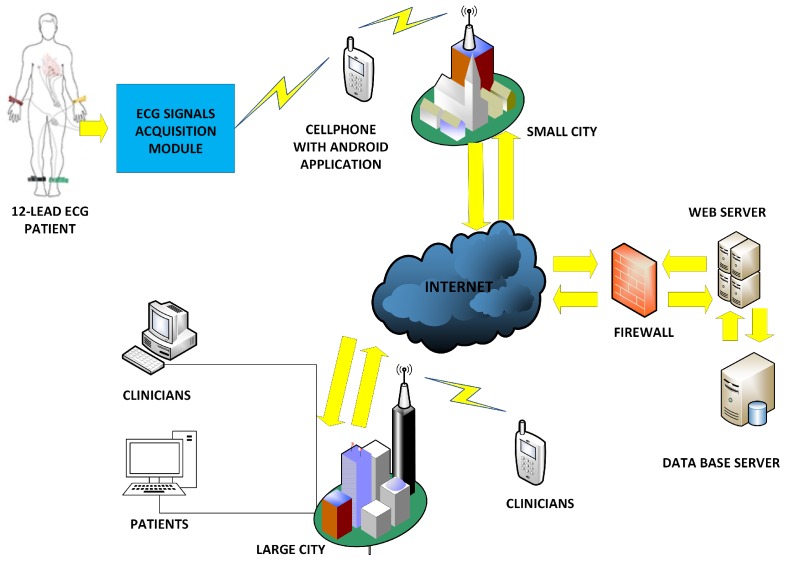
General diagram of the flexible 12-lead/Holter prototype hardware architecture, where large city and small city refer to urban and rural areas.

The general diagram proposed for the flexible 12-lead/Holter prototype is shown in Figure 1. This diagram shows an acquisition module following the usual approach of capturing biopotential signals from 10 electrodes on the patient body and then generating the 12-lead ECG from them, in the case of 12-lead use. This information is sent to a cellphone to be depicted for inspection. On the one hand, the application developed in the cellphone displays the ECG signals on the screen, and it stores them locally or sends them to the web server through the Internet. On the other hand, the application on the web server manages the navigation options for three types of user, namely, the patients, the doctors, and the system administrator. In addition, this application plots the real-time ECG signals received from the cellphone on the server and on the PC of those users that are registered into the system. Each of these modules are described with some detail in the following subsections.

### 3.1. Acquisition Module Hardware Architecture

**Figure 2:**
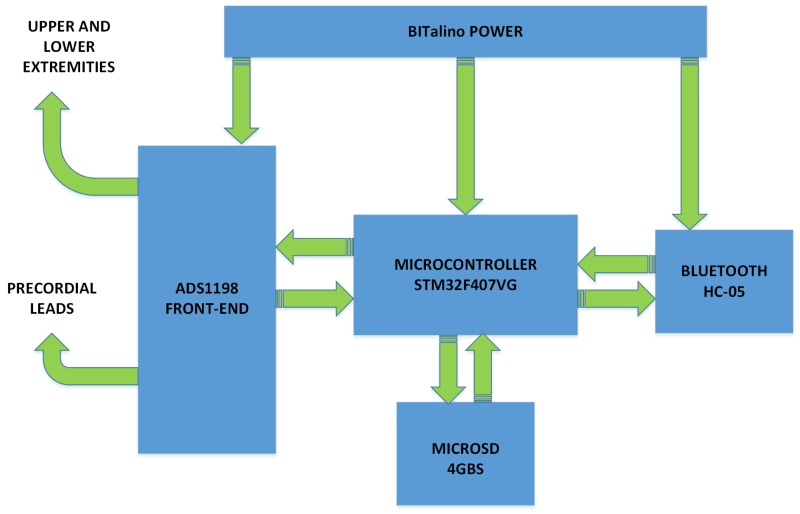
Block diagram of the acquisition module.

The ECG signal acquisition module receives biopotential signals from electrodes located on the patient body. Note that from a clinical viewpoint we need two cable sets, one with 10 and another with 3 cables, and they should be connected according to whether they are used as a 12-lead ECG or a Holter, to avoid floating cables when the Holter is used. On the other hand, one should keep in mind that Wilson’s central terminal, which is used as a reference potential in the 12-lead ECG, cannot be obtained from the three electrodes on the thorax in the Holter, so that the available leads are not directly translated from one system to another. This module achieves several relevant functions, namely, amplification, digitization, filtering, memory storage, and transmission to the cellphone. Figure 2 depicts a block diagram of the acquisition module.

The microcontroller is the fundamental component of the acquisition module, as far as this unit is responsible for controlling other devices, such as the Bluetooth module, the storage module, and the ADS1198. This microcontroller executes the following functions: providing the control signals to the ADS1198 for its initial programming, and handling the data reading of the eight channels obtained from the 10-electrode signals and the status registers; generation of the 12-lead ECG; handling the Bluetooth communication interface; handling the MicroSD interface for data storage; executing the processing algorithms for the compression of the ECG signals. Given that ECG signals are transmitted from this module to a cellphone, a compression algorithm based on modulated filter banks [25] is embedded into the module in order to reduce the data transmission rate. Thus, the system is allowed to work under two modes: the uncompressed mode, where the originally acquired ECG is sent with no processing, and the compressed mode, where the encoding system is applied.

The microcontroller selected for the acquisition module is the STM32F407VG, given that it has a 32-bit high performance M4 cortex ARM core with a floating point unit. This microcontroller works with a processing speed of 8 MIPS in the uncompressed mode and 168 MIPS in the compressed mode. The execution of the lossy compression algorithm requires several processes to decompose in subbands of the 1024-sample segments in a four-byte floating point. One of them is multiplication with the 192 × 16 analysis matrix, whose elements are in floating points, and this operation requires the microcontroller to execute the instructions in a lower time, which can be achieved by increasing its velocity to 168 MHz. Its architecture allows the execution of a 32-bit instruction for each clock cycle. The program memory of 1024 KB allows the storage of the program developed in Micro C. The program stores the coefficients of the analysis and synthesis matrices with 192 × 16 elements each. The availability of 192 KB of RAM in the microcontroller allows one to temporary store the convolution matrix required for the compression process. In this module, the ADS1198 integrated circuit was used for our implementation because it allows the generation of the 12-lead and it has 105 dB of Common Mode Rejection Ratio (CMRR) for each channel contributing to the noise elimination. The front-end for biopotential measurements in the ADS1198 has a 16-bit converter for each channel. This converter was set to work at 250 samples per second and per channel.

The ADS1198 included a sinc filter to attenuate the high-frequency noise. This sinc filter was configured with cut-off frequency of 125 Hz and with an overall sample rate of 2000 samples per second, which is equivalent to the 250 samples per second and per channel referred above. The analog input of the ADS1198 is fully differential. The STM32F407VG microcontroller uses the SPI for the control of ADS1198. Data recovery is done with the Continuous Data Reading (RDATAC) method, a method that allows the STM32F407VG to be read continuously from the ADS1198 without need to send operation codes. On the other hand, the ADS1198 yields a 19-byte data output, three control bytes, and 16 bytes of data corresponding to 2 bytes per channel (in binary two’s complement format).

**Figure 3:**
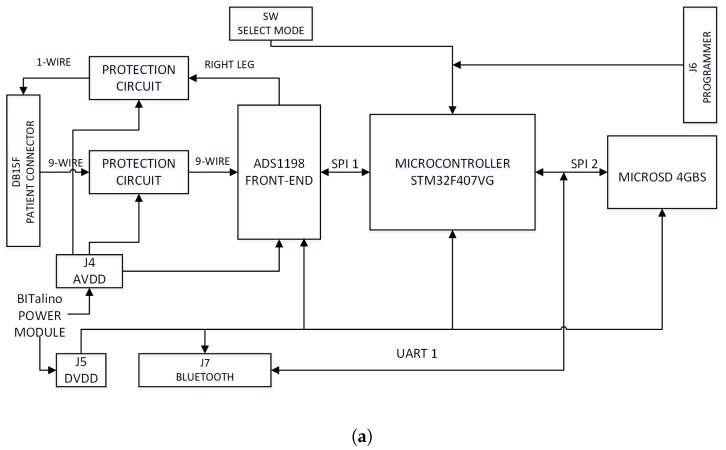

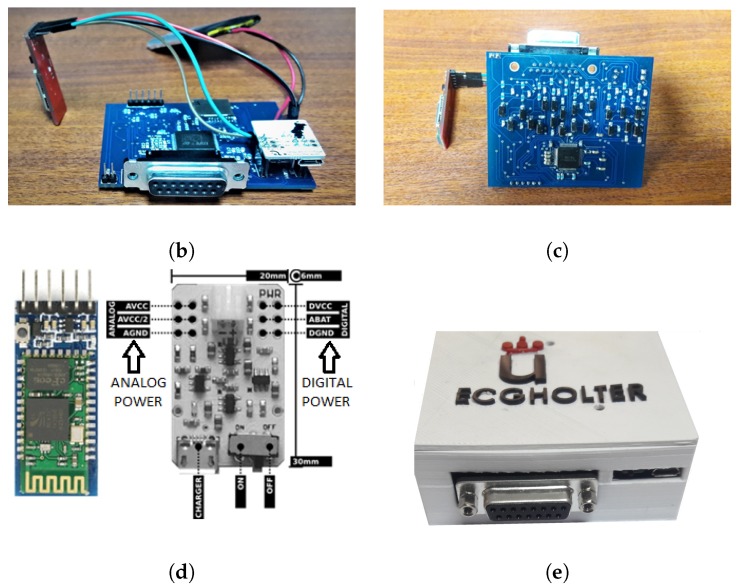
Acquisition module implementation: (**a**) Electronic circuit diagram. (**b**) Upper layer. (**c**) Lower layer. (**d**) Bluetooth module and BITalino™ power module. (**e**) Acquisition module mounted in a box. The electronic card was designed by considering the dimensions of the microSD memory support (15 mm × 17 mm) and the power module (20 mm × 30 mm). The dimensions of the shown electronic card which was designed for the acquisition module were 62 mm × 70 mm.

Our storage module was designed to include a MicroSD in this prototype with 4-GB capacity. It is controlled by the STM32F407VG microcontroller through the second SPI interface. The Bluetooth module is handled by the second Universal Asynchronous Receiver-Transmitter (UART) interface of the STM32F407VG microcontroller. The HC-05 Bluetooth module was programmed to manage the master mode with a bit rate of 8192 bits per second. With respect to the acquisition module, it was designed to work with a BITalino™ POWER module to provide the voltage levels required by the analog and digital circuits. The differentiation of the two power supply (analog for the ECG front-end and digital for the rest) is rather important for a cleaner recording, especially when radio frequency (RF) modules are used. This module is energized with a 3.7 V lithium battery with a charging capacity of 1000 mA/h. The module also has the capacity to control the battery charge trough a microUSB cable. The electronic circuit of the acquisition module (shown in Figure 3) was created using Proteus™ software.

The electronic card was designed by considering the dimensions of the microSD memory support (15 mm × 17 mm) and the power module (20 mm × 30 mm). The STM32F407VG microcontroller and the electronic components required for its operation (such as resistors, crystals, or capacitors) were placed on the upper side of the printed card, whereas the ADS1198 CI with the electronic components for its operation and for the patient protection was placed on the lower side of the printed card. The dimensions of the electronic card designed for the acquisition module, shown in Figure 3b,c, were 62 mm × 70 mm. Figure 3d show the Bluetooth module and the BITalino™ power supply module. A 3D-printed custom box was constructed to protect all the circuits, as shown in Figure 3e. The dimensions of the 3D printed custom box were 77 × 65 × 28 mm. The prototype had protection subsystems using Schottky diodes, to ensure patient safety when the equipment was connected to the power supply network.

### 3.2. Cellphone Module and Web Server Hardware Architecture

**Figure 4:**
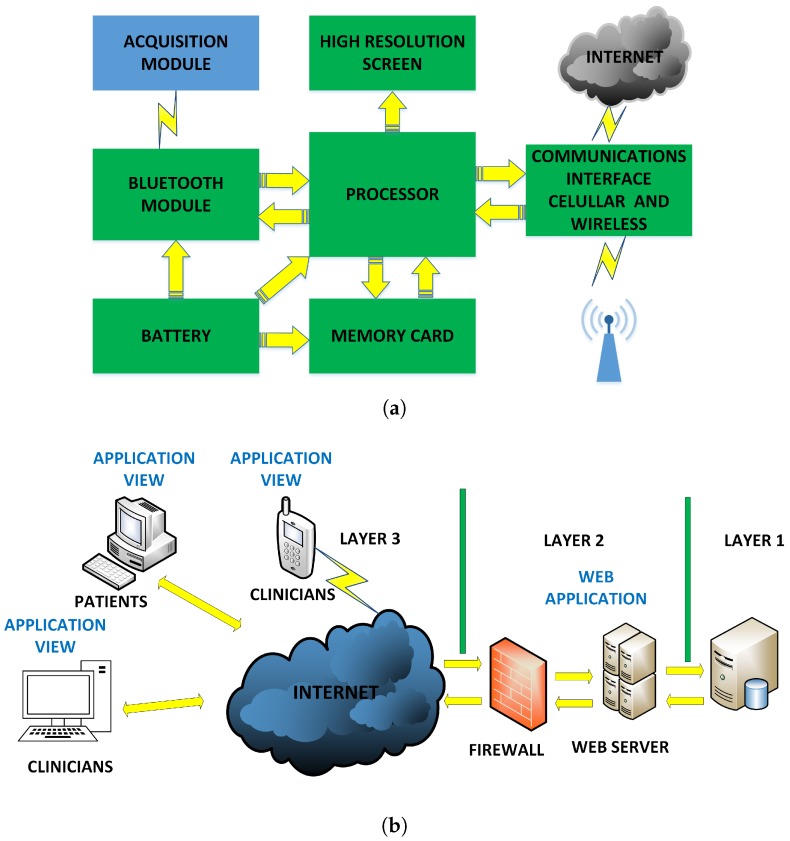
Hardware architecture: (**a**) Cellphone module. (**b**) Web server.

The purpose of the cellphone in the flexible 12-lead/Holter prototype is the visualization of the ECG leads received from the acquisition system via Bluetooth and their transmission to the web server via the Internet. Figure 4a shows the cellphone-module hardware architecture. This module was implemented with a cellphone powered by the Android™ operating system. The technical characteristics of the cellphone required for the application are a minimum screen resolution of 480 × 800, a minimum dual-core processor, a processing speed above 1.2 Ghz, the operating system Android™ OS v2.3 or higher, Wi-Fi 802.11 (a/b/g/n), and a 4-GB memory card.

The application layer requires an access server to host the telemedicine application software and to provide the cardiology specialist with access to the system. Figure 4b shows the web-server hardware architecture. As seen, the application server is a high processing capacity device that manages the database server to store the ECG records of the online and offline monitored patients. This architecture has a firewall to protect the data, as well as an Internet domain for remote use. Cloud services were contracted to a provider in order to professionally host the web-server application. The provider company offers the web hosting service with support for Java applications and high-capacity server technology according to the requirements of the web application, and it also provides the system with solid-state drives (SSDs) that increase the response speed without restrictions on access traffic.

## 4. Software Architecture of the Flexible 12-Lead/Holter Prototype

The software architecture of the flexible ECG/Holter prototype has three levels that are handled by their respective hardware. This section describes the applications at the microcontroller, at the cellular, and at the web application levels. All the software used in the higher (cellphone, web) and in the microcontroller levels of the prototype is free (see [26]). The application at the microcontroller level was developed with the Micro C software for ARM, whose license was acquired specifically for this work.

### 4.1. Acquisition Module Software Architecture

**Figure 5:**
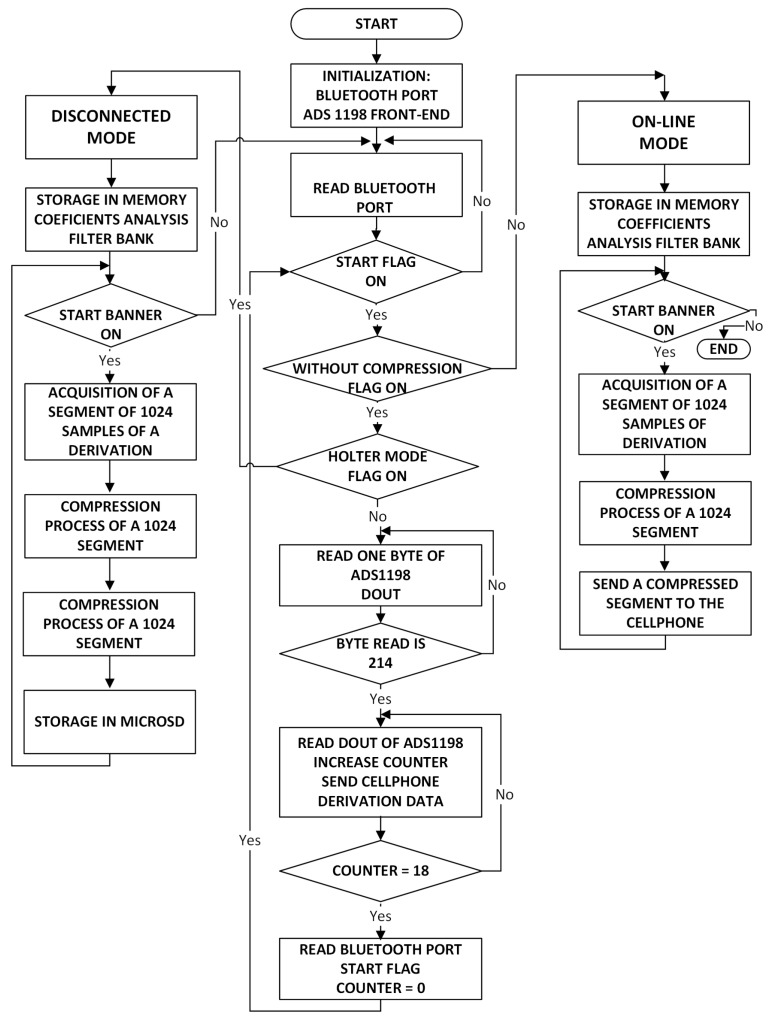
Application flowchart of the acquisition module.

The development software Mikro C PRO for ARM was used to program the software application of the acquisition module. The application contains the instructions that the STM32F microcontroller executes to manage the ADS1198 acquisition module, the storage in the MicroSD, and the Bluetooth module to communicate with the cellphone and to execute the compression algorithm. Micro C PRO is a low level software developed by MikroElektronika company for the family of ARM microcontrollers. This software was selected due to its versatility to handle the control ports of the ADS1198 IC, which perform the signal acquisition from the electrodes connected to the patient. The acquisition module application has mainly two operation modes, namely, online and disconnected modes. Figure 5 shows the application flowchart for both operation modes. The disconnected mode allows the acquisition module to work autonomously. Once this mode has been selected in the app, the acquisition module works autonomously without the cellphone. In this operation mode, the selected signals can be stored in the 4-GB MicroSD compressed or uncompressed (by default). The online mode allows one to send the signals to the cellphone via Bluetooth.

The evaluation of compressors was performed out in terms of compression capability and quality of the reconstructed signal [27]. The Compression Ratio (CR) informs of the bit reduction, and it is calculated according to $CR=\frac{bx}{bc}$, where bx and bc are the number of bits needed for the original and the compressed signal representation, respectively. The most usual parameter to measure the quality of the reconstructed signal is the Percentage Root-mean-square Difference (PRD), which is defined as $PRD=\sqrt{\frac{\sum_{1}^{N}{\left(x\left[n\right]-\hat{x}\left[n\right]\right)}^{2}}{\sum_{1}^{N}{\left(x\left[n\right]\right)}^{2}}}\times 100$, where x[n] is the original ECG signal and $\hat{x}\left[n\right]$ is the reconstructed one.

In order to compress the ECG signals, we implemented a filter bank technique, which uses criteria similar to those of MPEG1 to process the audio signals by dividing the working band into subbands. The technique of Nearly-Perfect Reconstruction Cosine Modulated Filter Bank (N-PR CMFB) was proposed in [28], and it was implemented here with an algorithm in the acquisition module program. The algorithm runs continuously with the 1024-sample ECG blocks that are obtained from the patient when the prototype works in compression mode. The algorithm performs the decomposition into 16 subbands to each 1024-sample input block with an N-PR CMFB, as well as the thresholding of the subband signals to guarantee the quality of the recovered signal. The implementation of an N-PR CMFB with *M* channels of maximum decimation where all the analysis and synthesis filters was obtained through the modulation of a low-pass prototype filter. The filter bank logic contains an analysis filter bank represented by an analysis matrix, which allows one to decompress the original signal in bands, and a synthesis filter bank, represented by a synthesis matrix. The coefficients of the analysis and synthesis filters were obtained in [25], starting with a low-pass filter prototype. The analysis and synthesis matrices had sizes 16 × 192, and they were used in the compression algorithm implemented in the software of the prototype acquisition module.

The target PRD was established a priori for this work to be 5%. The applied thresholding is dependent on the target PRD, as explained in [25]. Once the corresponding thresholded signal is obtained, the resulting samples are entropy-encoded as proposed in [25]. The significant coefficients are grouped and encoded with 8 bits per sample. A significance map is generated assigning ‘1’ to the significant coefficients and ‘0’ to the others. The map is then organized as a set of 8-bit integers. This map and the significant coefficients are stored or transmitted through the corresponding communication media.

### 4.2. Cellphone Software

The cellphone application was developed using the Eclipse™ software ( Ottawa, ON, Canada), which has a friendly graphical interface for developers. Eclipse is a generic application that allows the user to program in several languages. A plug-in for Android™ can be installed, and the SDK with the available Android™ (Baltimore, MD, USA) version can then be used. The application controls the acquisition module and graphics on the cellphone screen, and it runs the operation modes of the acquisition module through the buttons on the main screen of the graphic interface. If we are working without compression, the cellphone receives the information of the acquisition module, which is processed to separate each of the ECG signals from the 12-lead bitstream, and finally they can be continuously and simultaneously shown on the cellphone. The communication protocol used to transfer data via Bluetooth is formed by a 12 signed-integer array of 2 bytes, corresponding to the data of the 12 leads and the package header with identifier 241. The communication protocol to transfer data from the cellphones to the Internet uses a cloud package, consisting of a floating-point data matrix with 320 × 12 elements, where each column corresponds to a lead. The cellphone application converts into mV each time a lead data arrives from the acquisition module, shows it on the screen, and stores it in the corresponding position. Once the matrix on the cellphone application is full, data are sent to the web server. The generated traffic is 15,360 bytes per second. Each datum has a four-byte length. These 320 data are taken in 1.25 s, so the online transmission has a delay of 1.25 s. Note that this internal protocol is inserted inside the transmission control protocols ensuring secure and errorless wireless communication. In addition, the cellphone sends the information to the web server when the online button has been activated. If we are working with compression, the cellphone passes the compressed information from the acquisition module to the web server. In the disconnected mode, the cellphone configures the parameters of the operation module and then disconnects.

**Figure 6:**
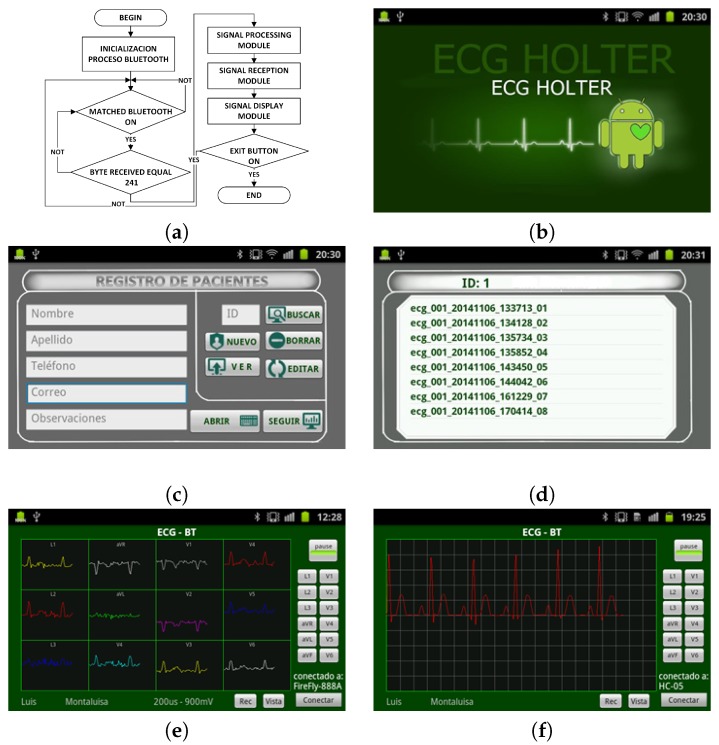
Smartphone software: (**a**) Patient data base field. (**b**) Home screen. (**c**) Patient registration screen. (**d**) Stored-record screen. (**e**) 12-lead ECG signal screen. (**f**) Single ECG signal screen.

This application has four modules: (1) the Bluetooth connection module, which establishes the communication between the cellphone and the acquisition module; (2) the ECG signal reception module, which performs the management of the data coming from the acquisition system; (3) the signal processing module, which here only performs the scale adjustment; (4) the display module, which calls the Eclipse™ display routines. Figure 6a shows an application flowchart. The graphical interface of the application represents the signals obtained in two modes, namely, the default mode, which shows one lead signal in the screen, and the connection mode, which displays the 12-lead ECG on the full screen. Figure 6e,f shows the 12-lead ECG on the full screen and the selected lead on the screen, respectively. The application has a local database to store patient information, and it assigns a unique identifier (ID) for each patient. Figure 6c shows the fields of the database. The recorded signal is stored in a plain file into a database on the mobile device memory, and the records can be viewed later from the registration list corresponding to each patient signals. The generated plain file conveys the patient ID, the date and time of the signal recording, and the sequential number of that corresponding signal. Figure 6d shows the register storage in the cellphone. The database is synchronized with the database of the web server when an online connection is made for patient monitoring purposes. The database allows one to search for the patient register. In addition, the application records the ECG signals for the required time periods and reproduces them on the cellphone screen under the user request.

### 4.3. Web Server Software

The web server application is responsible of providing online access for medical review. This application performs several front-end and back-end functions, namely, registration of user personal data, classification of user types, registration of clinical data from patients, user authentication, information management according to user permissions, display of performed examinations, and permanently available interface on the Internet.

The web server application uses the database server and the model view controller (MVC) for efficient management of remote users and database access. The database stores the clinical data and the 12-lead ECG signals from patients. The web server application was installed on the web server with the domain http://www.ecgholterurjc.com:8888/ECGBDD provided by the web hosting service. The application has two operation modes: (1) The uncompressed mode receives the 12-lead ECG signals information to depict them on the server screen or remote user screen. (2) The compressed mode receives the compressed information of one-lead ECG to plot it on the remote or local user screen.

**Figure 7:**
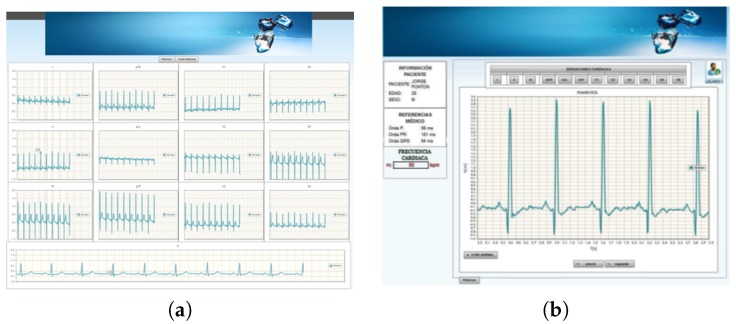
Web server application: (**a**) 12-lead ECG signals screen; (**b**) online display screen.

The application can display either the 12-lead ECG signals on the screen simultaneously or only one single ECG signal on the screen. Figure 7a,b show these two display modes of the ECG signals on the web application. The application has three forms of navigation according to the user type (doctors, patients, or managers). Each user has an account to access to the system, and these accounts are managed by the application administrator.

## 5. Experiments and Results

The technical validation process was carried out in four phases. In the first phase, the behavior of relevant external components was verified, in terms of storage memory and power supply battery. Tests were also carried out related to the system autonomy in terms of time limitations and storage capacity. The second phase of the technical validation process verified the quality of the signal acquired by the equipment without compression, to ensure that the designed electronics did not alter in any way the signal coming from the electrodes. In the third phase, the effects of the compression and decompression processes were scrutinized. In the fourth phase, a calibration of the prototype output was made by using a commercial electrocardiograph as a gold standard. The aim of this phase was to adjust the conversion equations to obtain a signal as similar as possible to the output of the commercial ECG. This technical validation of the 12-lead/Holter ECG prototype was carried out by comparing with the commercial electrocardiograph CardioExpress™ SL3 from SpaceLabs (Snoqualmie, WA, USA). The Minisim 1000 simulator from Netech™ (Grand Rapids, MI, USA) was also used to generate synthetic and controlled cardiac signals.

### 5.1. Experiment 1: Energy Autonomy and Storage Capacity Tests of the Prototype

Energy autonomy refers to the time that the device keeps working with the payload of a battery. The LIPO-type battery (lithium-polymer) used in the proposed mobile prototype has a 1000 mAh capacity and a 3.7 V nominal voltage. The autonomy test determined the maximum operating time of the prototype, working from the battery at full charge (4.52 V) until it reached its minimum usable voltage (3.3 V), given that the BITalino™ power module requires a minimum input of 3.3 V to deliver an analog voltage of 3.3 V and a digital voltage of 3.3 V at its output.

**Figure 8:**
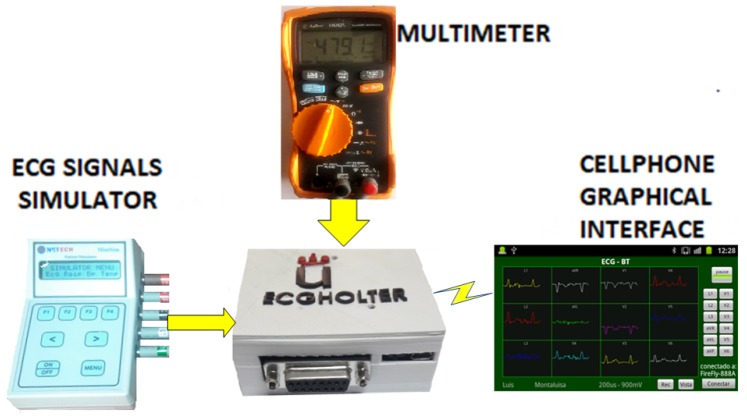
Scenario of Experiment 1 for the battery charge-duration test.

The setup scenario implemented for the autonomy validation test is shown in Figure 8. It can be seen that the 12-lead/Holter ECG prototype acquired signals from the ECG simulator and it sent them via Bluetooth to the cellphone. The test was first performed without compression (lower computing load for the microcontroller working at 8 MHz) and then with signal compression (working at 168 MHz). The battery terminals were connected all the time to a voltmeter to verify when the minimum usable voltage was reached.

**Figure 9:**
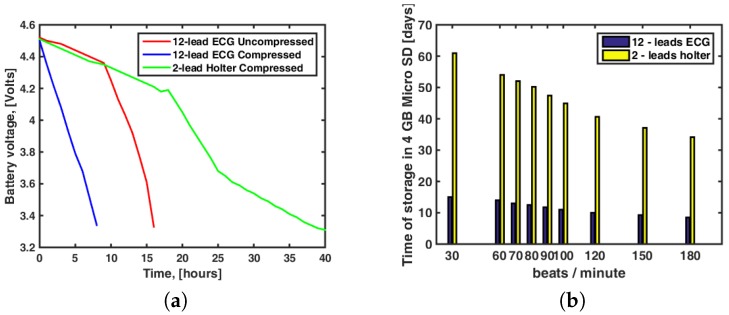
Results of Experiment 1: (**a**) Energy autonomy tests. (**b**) Storage capacity tests.

The results of the autonomy tests for the uncompressed and compressed modes with 12-lead ECG use, as well as with Holter use, are shown in Figure 9. Additional considerations need to be made in terms of the energy consumption. In the uncompressed mode, the microcontroller works at 8-Mhz frequency with an average consumption of 20 mA, the ADS1198 has a consumption of 2.3 mA, the HC-05 Bluetooth module has a consumption of 25 mA, and the microSD has a consumption of 8 mA. Hence, the total theoretical current consumption is 55.3 mA, whereas the total measured current consumption is 59.58 mA for this mode. In the compressed mode, the microcontroller works at 168 Mhz with an average consumption of 93 mA, and the other components have a consumption similar to the uncompressed mode. The total theoretical current consumption is 128 mA and the total measured current consumption is 110 mA for this mode. The autonomy times were 16 h for the uncompressed mode and 8 h and 28 min for the compressed mode using 12 leads, and it was 40.5 h with the Holter use (microcontroller working at 8 Mhz with a total measured current consumption of 29 mA). Note again that the present prototype did not address the battery optimization, so the use of higher capacity batteries will notably enlarge the system energy autonomy.

We conducted specific storage benchmarking with different heart rhythms, both for the 10-electrode (12-lead) mode and for the 3-electrode (2-lead) Holter mode. As seen in Figure 9b, for a typical cardiac frequency of 80 bpm, the 12-lead mode can store in the microSD memory up to 4 GB of compressed continuous recording during 12.5 days. Also, for the Holter mode with 2 leads we can store up to 50 days of continuous recording. The prototype supports, without need of any hardware or software modification, its expansion up to 32 GB, hence this storing capacity can be readily increased and multiplied by four.

### 5.2. Experiment 2: Validation of Signal Compression and Decompression

**Figure 10:**
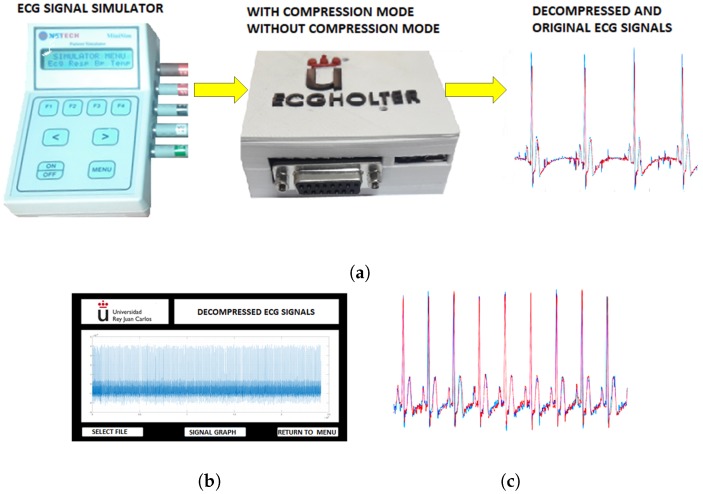
Experiment 3 for validation of signal compression and decompression: (**a**) ECG signal quality tests with compression and decompression processes. (**b**) ECG signal after the compression and decompression process. (**c**) The original uncompressed signal and the example signal when compressed and decompressed by the prototype.

Once verified the acquisition quality, we also verified the analog-to-digital conversion, the Bluetooth quality for sending to and reception from the cellphone, and the prototype representation system. Figure 10a shows the test-bed used to perform the validation of the compression and decompression processes. The aim here was to obtain the same signal and to save it both in a compressed file and in an uncompressed file. Later, both files were extracted from the prototype, and after decompression, both were compared graphically and through the calculation of the PRD of both signals.

The ECG signal for this test was a non-pathological simulated signal of NSR, with a frequency of 70 bpm. The prototype performed the compression process using the technique based on nearly perfect reconstruction cosine modulated filter banks, configuring them with a theoretical PRD of 5%. The results obtained after the decompression process on the graphic interface of the computer are shown in Figure 10b. In Figure 10c, a segment of 1024 samples of the original ECG signal is plotted without compression (in blue), and after compressed and decompressed by the system (in red) for the same segment. The two signals have highly similar characteristics and the real PRD obtained by comparing them was 5.2%, very close to the one defined by the system. These same signals, but with one-hour duration periods, were used to calculate the compression ratio of the system (configured for a theoretical PRD of 5%).

The process of calculating the compression ratio was performed with non-pathological synthetic signals with different cardiac rhythms. One-hour captures were made and saved with and without compression. These files were extracted from the device to verify their size. Table 2 shows the results obtained with this test and the calculated compression ratio. The compression rate for an NSR that can be considered as normal (80 bpm) was 16.85. An additional test was performed for the sinus arrhythmia pathology generated by the base frequency simulator of 80 bpm, obtaining a compression ratio of 16.25.

### 5.3. Experiment 3: Calibration with Commercial Electrocardiograph

**Figure 11:**
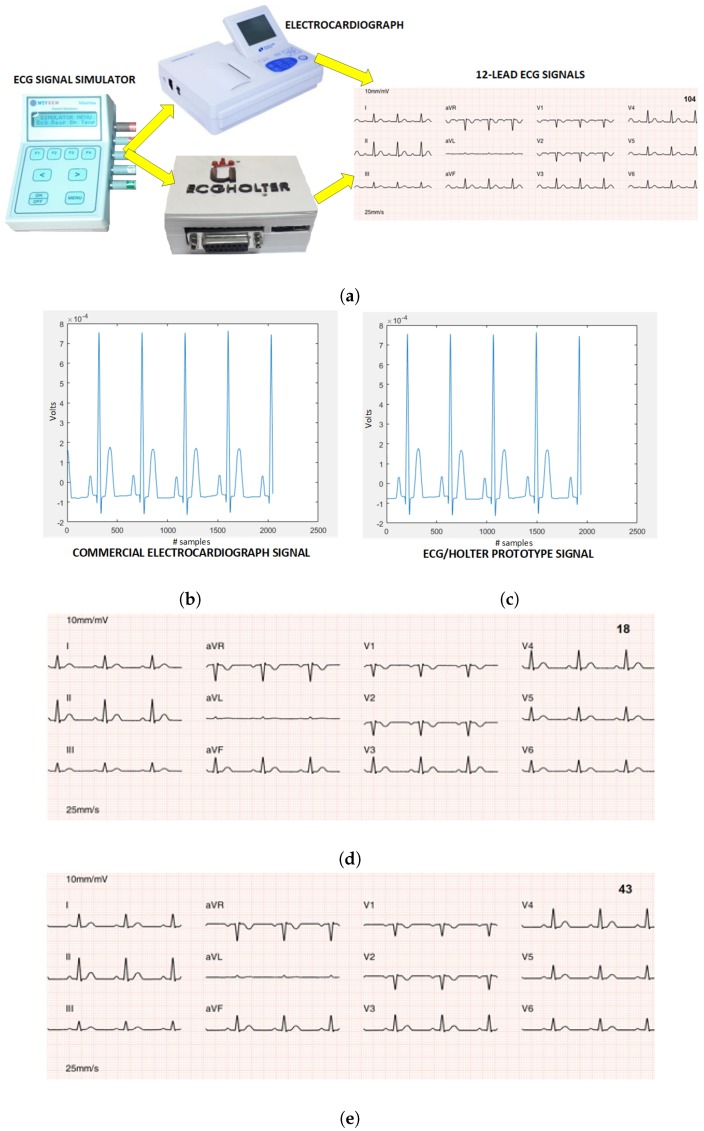
Experiment 4 for prototype calibration with respect to commercial electrocardiograph: (**a**) Calibration scenario of the 12-lead/Holter ECG prototype. (**b**) D2 lead reference signal of commercial electrocardiograph. (**c**) D2 lead calibrated signal of 12-lead/Holter ECG prototype (right). (**d**) 12-lead ECG signals obtained with the prototype. (**e**) 12-lead ECG signals obtained with the commercial electrocardiograph.

Finally, in the fourth phase of the technical validation, the prototype was calibrated by taking the commercial electrocardiograph as the reference standard. Figure 11a shows the calibration scenario of the 12-lead/Holter prototype. The first signal used for calibration was a non-pathological synthetic ECG signal with a rhythm of 70 bpm, which was compressed by the prototype to a theoretical 5% PRD. The process consisted in simultaneously capturing the signals of the ECG simulator with the 12-lead/Holter prototype and with the commercial electrocardiograph. The signals were stored in the MicroSD memory of the prototype and in the flash memory of the electrocardiograph, respectively. Both signals were downloaded to a computer to be plotted in the same way. Gain adjustments and conversion equations of the integrated ADS1198 were carried out until the maximum similarity was achieved for the signals from the prototype and from the commercial equipment. Figure 11b,c show D2 lead signal used as a calibration standard of the commercial electrocardiograph and the same obtained from the prototype after having proceeded with its calibration.

As can be seen, both test signals are indistinguishable. A technical comparison was made by calculating their PRD, obtaining 0.01%. The plotting and printing of the signals was done using specifically designed software to reproduce the format used by the commercial electrocardiograph, which included the 12 leads. The 12-lead signals obtained with the prototype and with the commercial electrocardiograph, respectively, are shown in Figure 11d,e.

## 6. Clinical Tests on the ECG Waveforms

After all the previous tests for technically validating the system and after we had obtained a format making indistinguishable the ECG capture and representation procedures (prototype versus commercial equipment), we addressed a final blind clinical test on the ECG waveforms about the quality of the obtained signals according to the end-user in electrocardiology. These clinical tests on the ECG waveforms were performed using both synthetic signals and real patient signals. It is fundamental to highlight that this validation process is not a clinical trial as such, but instead a prior quality evaluation in order to verify if a larger trial should be conducted in a large population in the best possible system conditions.

### 6.1. Experiment 4: Acquisition of ECG Signals from Patients

In the city of Quito, specifically at Pablo Arturo Suárez Hospital, several ECG acquisitions were carried out over a week, both with the prototype and with the commercial electrocardiograph, in 22 patients who attended to the cardiology service due to suspected cardiac pathology. All the participants were informed and agreed to participate in the study, which was conducted in accordance with the Declaration of Helsinki and had been previously approved by the Hospital Ethics Committee with reference 17/11/2016.

Figure 11a (Experiment 4) shows the test scenario for clinical validation with real patients. The prototype recordings were saved both in compressed and uncompressed modes. Then, 66 ECG signals with 12-leads each were generated in a similar format to the presented by the commercial electrocardiograph on thermal paper, as shown in Figure 11a. Patients did not notice the change from one system to another (prototype vs. commercial equipment) since the same electrodes were used in both captures (both systems used the same DB15 connector).

### 6.2. Experiment 5: Acquisition of ECG Signals from the Simulator

**Figure 12:**
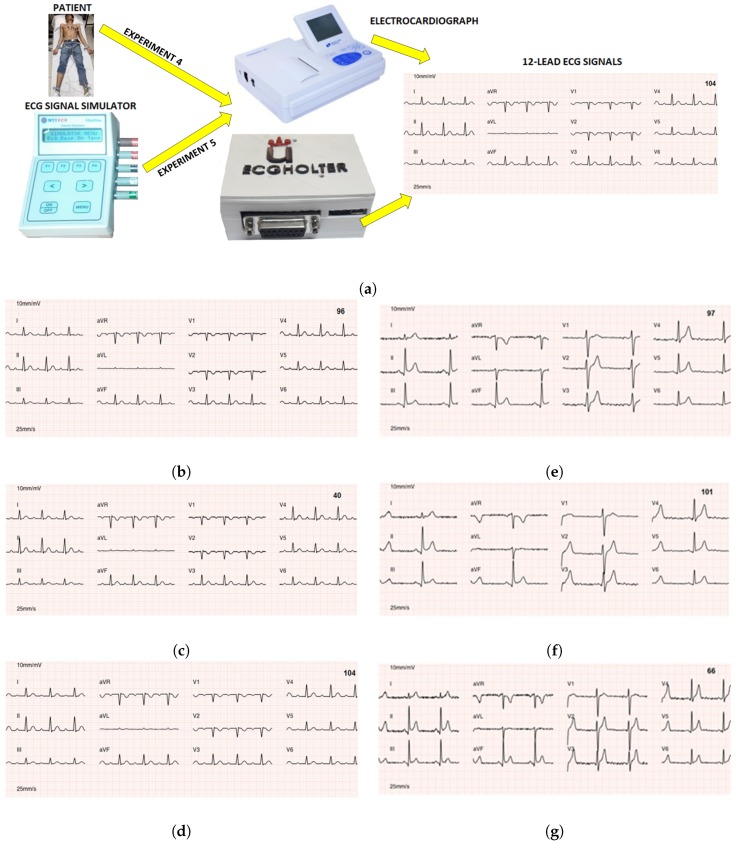
Experiments 4 and 5: (**a**) Test scenario for clinical validation with real patients and with ECG simulator. The ECG signals presented to physicians specialized in cardiology are also shown—in signals from the simulator with AB1 pathology (left) and from patients (right). (**b**,**e**) ECG signals obtained with the prototype from simulator and the patient, and without compression. (**c**,**f**) The same obtained by the prototype with compression. (**d**,**g**) ECG signals obtained by the commercial electrocardiograph.

To the previously described 66 recordings from real patients, we added another 66 synthetic recordings obtained from the Netech Minisim 1000 simulator. The system was configured to emulate 22 cardiac pathologies and different cardiac rhythms. These signals were obtained with the commercial electrocardiograph and the prototype with compression and without compression, as can be seen in Figure 12a (Experiment 5). The obtained files (from the commercial equipment, from the prototype without compression, and from the prototype with compression) were stored so that a computer could present the files in the same format (as shown in the previous section) that commercial systems usually present them.

### 6.3. Experiment 6: Tests on ECG Waveforms by Cardiologists

The 132 ECG signals (66 × 2) obtained in the previous sections with 12 leads each were each stored in an image file each and labeled with a random number (1 to 132). Figure 12b–g show an example of the ECG signals presented to cardiologists. The clinical tests on the ECG waveform consisted of showing these 132 ECG to three different cardiologists (one from the Community of Madrid and two from Murcia, Spain). A table with 132 rows was attached to these files for the cardiologist to issue his diagnosis. The objective was to verify whether each cardiologist issued the same diagnostic in the ECG of the same patient, independently of the ECG origin.

The evaluation protocol followed by the cardiologists for ECG waveform testing started by determining whether the ECG was well performed or not. The paper speed and amplitude were then checked to be normal; for instance, in a standard ECG the speed was 25 mm/s and the amplitude 1 mV per 10 mm. The analysis subsequently included the following points: cardiac frequency calculation, cardiac rhythm analysis, evaluation of PR interval, evaluation of QT interval, electric axis, alterations in the ST segment, and other electrocardiographic alterations.

Table 3 shows a summary of the diagnostic issued by cardiologists. *Agreement* was considered when a cardiologist agreed in the diagnostic for the same signal taken with the prototype in the uncompressed mode, with the prototype in compression mode, and with the commercial electrocardiograph. If there were differences between the uncompressed signal of the prototype and the one of the commercial standard, or between the compressed signal of the prototype and the one of the commercial standard, it was considered disagreement. At this point, the percentage of agreed cases was 92.42%, with a total of 10 cases recording some disagreement among the cardiologists. One of the cardiologists was asked to analyze the disagreed cases, aiming to determine if these were due to issues with the simulator equipment, in the prototype capture, or in the recording conditions with only three beats. Therefore, all the newly revised recordings included exactly the same time duration and number of beats, as well as an additional strip of Lead 2 with 10 s of ECG. Up to 8 of these 10 cases were found to be consistent. These results provided evidence that the three sources are clinically usable for diagnostic purposes. A word of caution was expressed by the cardiologists: this set of cases did not include examples of subtle electrocardiographic changes, such as epsilon waves, fragmentation or microfragmentation, preexcitation, and mild elevations or depressions of the ST segment. These cases are not necessarily usual but can certainly be present in the clinical daily practice, so they should be considered. Hence, a larger-scale trial could be addressed, while taking into account the issues highlighted in the present work.

## 7. Discussion and Conclusions

In this paper we presented the design and implementation of a flexible 12-lead/Holter ECG prototype with visualization interface in the cellphone and remote transfer to a web server for remote access. The prototype has the ability to function in a flexible form as a Holter and as an electrocardiograph thanks to the designed hardware architecture, and providing the prototype with the ability to deliver ECG signals with high clinical quality. Our system is flexible enough to combine the processing power of the prototype, with that of the cellphone and that of the remote server in order to properly perform compression processes such as (we have implemented and checked, inside the prototype, compression algorithms based on modulated-cosine filter-banks) adaptive-band filtering, baseline wander and artefact cancellation, multichannel decomposition with principal component analysis or independent component analysis, feature extraction, delineation analysis, and arrhythmic event detection. In our system, although the compression algorithm introduces information loss and modifies the waveform of the reconstructed wave, the diagnostic information is still preserved with the advantage of an increased compression ratio of around 15.

*On the Technical Achievements.* The experiments carried out made it possible to determine its efficiency in energy autonomy, which in combination with large storage capacity allow its operation as a Holter for several days. Nevertheless, the execution of the compression algorithms has a high computational cost for the microcontroller, causing a notably high consumption of battery current affecting the autonomy of the system, though time usage could be logically increased by providing higher capacity batteries. In addition, a commercial prototype should allow the recharge of the system in use, without risks for the patient, an end that has not been implemented in this prototype because it goes beyond the scope of the present validation. In order to ensure the clinical quality, prototype calibration experiments were carried out by taking a commercial electrocardiograph model as a reference. The experimentation with signals obtained from real patients and with synthetic signals, together with their corresponding medical validation, generated high agreement among the cardiologists (98.48%). The price of this flexible 12-lead/Holter prototype was around 90 dollars, obtaining clinical quality for diagnosis, with a minimum compression ratio of the signals of 14.35. In [29], it was mentioned that the medical format protocols use algorithms that usually execute lossless compression techniques to compress the ECG signals in order to guarantee the quality of the signals. Alternatively, in the present work, we have verified that it is possible to use conservative lossy-compression techniques while still ensuring the signal quality, significantly reducing the recording sizes and the bandwidth needed to send signals through narrow communication channels, such as those ones that are found in rural areas of developing countries.

*On the System Flexibility.* It has to be kept in mind that there is a clear clinical difference between the ECG recording system with a double use, namely, the short-term 12-lead ECG recording in controlled environments and at rest, and Holter ECG recording in ambulatory use. Our aim here was to create a flexible system in terms of the possibility of using a single device in rural and isolated areas of developing countries, so that a single device can provide both, according to the clinician’s or nurse’s need. Recall that it is uncommon to use a Holter system with 12 leads for Holter long-term monitoring, because the need for wires would make it impractical and the gain in information retrieved with respect to the 2–3 lead system does not compensate in general the effort. In some clinical scenarios, a 12-lead long-term Holter could be useful, for instance, in order to document the morphology of extrasystoles or spontaneous ventricular tachycardia (which can be clinically relevant in some cases) or to document regional elevations of ST-segment that can be visible only in some leads, but this should be useful in only a very small percentage of the usual indications for the Holter.

*Limitations of the Current Development.* Several limitations must be mentioned. A signal quality validation on ambulatory conditions, which is crucial for its final use, is pending. The same can be said for a suitable and definitive clinical validation of the system, which is beyond the scope of the present work. Our small-scale validation did not reach a multicenter evaluation or certification, but it was oriented toward this direction, aiming to overcome this limitation of some proposals of mobile ECG devices in the literature. It is necessary to include records with subtle electrocardiographic changes among those cases to review, in order to verify that the filters or the compression and decompression algorithms do not mask epsilon, fragmentation or pre-excitation waves, or mild elevations or depressions of the ST segment. Nevertheless, the current prototype has put together a new scenario for mobile ECG systems by taking advantage of currently available technology and knowledge.

*Clinical Usefulness.* From a clinical point of view, several clinical needs can be addressed with the proposed prototype. First, a conventional 12-lead ECG could be made at home, still needing to be at rest and with very controlled conditions, and it could be subsequently transmitted by the nurse through the mobile to the clinician in a domiciliary attention service, which is still a low-developed commercial scenario. Second, in an emergency scenario where the general clinician is willing to make an ECG and consulting it with the cardiologist, this can be done nowadays from many centers and small hospitals, but it is not often implemented in rural areas of developing countries. In both cases, 12-lead ECG availability is necessary. Third, the application can be used to detect asymptomatic or infrequent arrhythmias, and in these cases, the patient carries the device in order to transmit the ECG to the clinician either when symptoms happen or on a scheduled basis. This kind of diagnosis is usually focused on arrhythmia detection, and it is widely accepted that a single lead is not enough. Two leads is usual, and three is better. More leads are scarcely used in this scenario. On the other hand, if the patient’s comfort is compromised, it is easier for the patient to put two fingers on the mobile or press two electrodes on her/his chest, rather than using 10 surface leads. There is a variety of devices of this type, from recent mobile systems such as Kardia™ to implantable Holters. The current system is especially flexible in this scenario. Finally, our system can be useful for recording a 12-lead ECG for an extended period of time (24 h or more), this being equivalent to a combination of 12-lead and Holter. Most of the patients referred to a standard Holter (24–48 h) are suspected of altered rhythm, but in general 2–3 leads are enough, and some patients will benefit from more leads, such as patients with thoracic recurrent pain with suspected ischemia for coronary spasm and patients with low-frequency ventricular ectopy, in which we are interested in the detailed morphology of the premature beats.

*From the Prototype to the Medical Device.* In our study and in others related to mobile ECG systems, it should be noted that mobile cellphones are hard to regulate as clinical devices, although software can actually be regulated as such. At this moment, the present work stays at the prototype stage, as many others in the literature. Nevertheless, our motivation is clearly supported by the fact that rural regions of developing countries have limited availability to electrocardiography equipment, due to the high cost of commercial equipment. However, the widespread use of cellphones in the population makes them an attractive option for many health applications, which makes it worth pursuing the usually hard and long effort to pass the regulation stages such as FDA approval. A clinical validation of the system would require one to check its reliability in different pathologies, and this could be hard and complex due to their variety. Those pathologies with clear electrocardiographic repercussion (such as evident morphological changes in the ECG, a clear increment of wave duration, and the appearance or disappearance of evident deflections) would not represent an issue, as they reproduce similar phenomena to the ones analyzed in a normal ECG. Those ones generating subtle changes (such as doubtful preexcitation, a slight deviation of ST-segment, or small Q waves) should be the ones to be checked in a wider clinical study. On the other hand, the technical standards that are usually required for medical devices include such diverse aspects as patient security, resistance to physical agents, sampling rate, filtering, device documentation, benchmarks to be performed, and electromagnetic compatibility. All of them are included in specific documents from the regulatory agencies (FDA in the USA, and several in the European Union), see for instance [30] for the 12-lead ECG and [31] for the Holter. These are hot topics for companies in the electromedicine field, though they are currently not part of the prototype state in this paper.

*Usefulness in Rural Areas.* In [2,17], it was pointed out that one of the problems in rural areas is the bandwidth limitation and the subsequently needed bit-rate reduction of the information flow. In these environments, the use of the developed prototype would be advantageously applicable. In rural areas of developing countries, the access to mobile broadband services (3G or higher) is always limited. In 2015, the International Telecommunications Union (ITU) reported that 71% of the rural population did not have 3G service [32]. In 2016, the ITU indicated that only 67% of the world’s rural population had access to this broadband service [33]. Approximately 450 million people living in rural areas do not have mobile coverage [34]. In regions with high inequality such as Latin America, the situation differs greatly from one country to another. In Cuba and Nicaragua, no more than 1% penetration is reported, Paraguay reports 4% and Peru, Honduras, and Bolivia reported respectively 14%, 16%, and 28% according to the data of the ITU and the GSMA of 2015 [35]. Therefore, it is necessary to use compression techniques that guarantee a high compression ratio of the signals transmitted by these communication systems.

The results achieved in the prototype development and in the experimentation allow us to conclude that it has been verified that the high compression rates achieved with compression algorithms with losses guarantees a high clinical quality when a robust and low-cost hardware structure is available.

## Figures and Tables

**Table 1:** Comparative table of recent and related works.

	Proposed	[18]	[11]	[19]	[20]	[21]	[14]	[22]	[23]	[24]
Number of leads	12	1	1	1	1	1	12	1	1	12
Use of Cellphone	Yes	Yes	Yes	Yes	No	Yes	Yes	Yes	Yes	No
Parameter detection	No	Yes	Yes	Yes	Yes	No	No	Yes	Yes	No
Use of Internet cloud	Yes	No	No	No	No	No	No	No	Yes	Yes
Compression capacity	Yes	No	No	No	No	No	No	No	No	No
Power cons. (mW)	120	150	140	160	180	150	150	150	150	150
CMRR (dB)	105	80	90	70	80	80	80	80	80	80
Noise (uVpp)	12	20	15	22	20	20	20	20	20	20
Input imp. (Siemens)	10	2	2	2	2	2	10	2	2	10
Sampling rate (Hz)	125	40	40	60	60	60	100	80	80	100

**Table 2:** Compression ratio for NSR signal with different rhythms.

Prototype Action	Heart Rate	Uncompressed File Size	Compressed File Size	Compression Ratio
Test 1	30 beats/min	4.227 MB	0.206056 MB	20.48
Test 2	80 beats/min	4.227 MB	0.250432 MB	16.85
Test 3	120 beats/min	4.227 MB	0.263160 MB	16.035
Test 4	180 beats/min	4.227 MB	0.294120 MB	14.35

**Table 3:** Tabulated results with the tests performed with synthetic and real signals.

Clinical Criteria	Cardiologist 1	Cardiologist 2	Cardiologist 3	Total
Agreement	37	43	42	122
Disagreement	7	1	2	10
Percentage of agreed cases over ECG total				92.42%
